# Targeting EphA2 in Bladder Cancer Using a Novel Antibody-Directed Nanotherapeutic

**DOI:** 10.3390/pharmaceutics12100996

**Published:** 2020-10-20

**Authors:** Walid Kamoun, Elden Swindell, Christine Pien, Lia Luus, Jason Cain, Minh Pham, Irawati Kandela, Zhaohua Richard Huang, Suresh K. Tipparaju, Alexander Koshkaryev, Vasileios Askoxylakis, Dmitri B. Kirpotin, Troy Bloom, Mari Mino-Kenudson, James D. Marks, Alena Zalutskaya, Wiam Bshara, Carl Morrison, Daryl C. Drummond

**Affiliations:** 1 Merrimack Pharmaceuticals, Inc., Cambridge, MA 02142, USA; wskamoun@hotmail.com (W.K.); epswindell@gmail.com (E.S.); christinepien@yahoo.com (C.P.); lluus3@gmail.com (L.L.); jasoncain25@gmail.com (J.C.); minhthu671@gmail.com (M.P.); zhaohua.huang@gmail.com (Z.R.H.); tsuresh_kumar@yahoo.com (S.K.T.); akoshkaryev@akageramed.com (A.K.); v.askoxylakis@gmail.com (V.A.); dkirpo@earthlink.net (D.B.K.); troy.bloom@comcast.net (T.B.); zalutskaya@gmail.com (A.Z.); 2 Developmental Therapeutics Core Facility, Northwestern University, Evanston, IL 60208, USA; i-kandela@northwestern.edu; 3 Massachusetts General Hospital, Harvard Medical School, Boston, MA 02115, USA; mminokenudson@partners.org; 4 Departments of Anesthesia and Pharmaceutical Chemistry, University of California at San Francisco, San Francisco, CA 94110, USA; Jim.Marks@ucsf.edu; 5 Roswell Park Cancer Institute, Buffalo, NY 14203, USA; wiam.bshara@roswellpark.org (W.B.); carl.morrison@omniseq.com (C.M.)

**Keywords:** bladder cancer, EphA2, antibody directed nanotherapeutic, liposomes, immunoliposomes, nanocarrier

## Abstract

Ephrin receptor A2 (EphA2) is a member of the Ephrin/Eph receptor cell-to-cell signaling family of molecules, and it plays a key role in cell proliferation, differentiation, and migration. EphA2 is overexpressed in a broad range of cancers, and its expression is in many cases associated with poor prognosis. We recently developed a novel EphA2-targeting antibody-directed nanotherapeutic encapsulating a labile prodrug of docetaxel (EphA2-ILs-DTXp) for the treatment of EphA2-expressing malignancies. Here, we characterized the expression of EphA2 in bladder cancer using immunohistochemistry in 177 human bladder cancer samples and determined the preclinical efficacy of EphA2-ILs-DTXp in four EphA2-positive patient-derived xenograft (PDX) models of the disease, either as a monotherapy, or in combination with gemcitabine. EphA2 expression was detected in 80–100% of bladder cancer samples and correlated with shorter patient survival. EphA2 was found to be expressed in tumor cells and/or tumor-associated blood vessels in both primary and metastatic lesions with a concordance rate of approximately 90%. The EphA2-targeted antibody-directed nanotherapeutic EphA2-ILs-DTXp controlled tumor growth, mediated greater regression, and was more active than free docetaxel at equitoxic dosing in all four EphA2-positive bladder cancer PDX models. Combination of EphA2-ILs-DTXp and gemcitabine in one PDX model led to improved tumor growth control compared to monotherapies or the combination of free docetaxel and gemcitabine. These data demonstrating the prevalence of EphA2 in bladder cancers and efficacy of EphA2-ILs-DTXp in PDX models support the clinical exploration of EphA2 targeting in bladder cancer.

## 1. Introduction

Urothelial carcinoma of the bladder is diagnosed in approximately 80,000 patients each year in the United States, and leads to 17,000 deaths annually [1]. Although local therapies can be effective at controlling non-muscle invasive bladder cancer, 20–30% of patients’ disease recurs and progresses to advanced stages, including locally advanced and metastatic disease. Despite the emergence of novel therapeutic modalities improving outcomes in certain subsets of patients, the overall five-year survival rate for patients with metastatic bladder cancer remains low at ~5% [2].

Platinum-based conventional chemotherapy in the neoadjuvant setting has shown promising activity in the locally invasive setting, but the benefit has been more limited in metastatic disease [2]. Moreover, patients with metastases who relapse after first line chemotherapy have very few treatment options [3]. Small Phase II trials suggested that taxanes (paclitaxel (PTX) and docetaxel (DTX)) can be reasonably active in the second line setting, especially when combined with gemcitabine, or when used as a triple combination with gemcitabine (Gem) and carboplatin (CBDP) [4,5], reaching objective response rates of around 30–60% for PTX + Gem [5], 47% for DTX + Gem [4], and 54% for DTX + Gem + CBDP [6]. Recently, there have been significant efforts towards identifying novel subtypes of bladder cancer and using these subtypes to design targeted treatments [7,8]. Immune checkpoint inhibitors have shown promise in bladder cancer, especially among patients with high expression of PD-L1/PD-1, leading to the approval of PD-L1 and PD-1 targeted therapies in second and cisplatin-ineligible first-line therapy settings [7,9,10,11]. However, there is accumulating evidence that the immune checkpoint blockade is not always sufficient to control tumor progression [12], underscoring the need for development of novel, more efficacious treatments.

Ephrin type A receptor 2 (EphA2) is a receptor tyrosine kinase that regulates cell–cell interaction, angiogenesis, and differentiation [13]. EphA2 overexpression in cancer cells leads to a ligand-independent increase in oncogenic signal transduction. In addition to bladder cancer [14], EphA2 overexpression is observed in numerous indications, including non-small cell lung cancer (NSCLC) [15], breast cancer [16,17], gastric cancer [18], esophageal cancer [19], pancreatic carcinoma [20], colorectal cancer [21], prostate cancer [22], head and neck cancer [23], glioblastoma [24], and ovarian cancer [25]. EphA2 expression in patients with bladder cancer is prevalent and is associated with later stage metastatic disease [14]; however, its prognostic relevance remains largely unclear.

EphA2 is membrane bound and, therefore, accessible to targeted drugs [26]. Moreover, it is readily internalized upon antibody binding [26]. These properties make EphA2 a promising candidate for the development of targeted cytotoxic drugs. Several EphA2-targeted agents that aimed to deliver cytotoxic molecules were developed including antibody drug conjugates (ADCs) [27], agonist peptide-drug conjugates [28,29], and targeted-nanotherapeutics [30]. While these studies provided evidence of enhanced preclinical in vivo activity in primary and metastatic cancer models, mechanisms linking EphA2-targeting with increased efficacy were not fully elucidated. Moreover, the only EphA2-targeted ADC that reached the clinical phase of development, MEDI-547, showed significant toxicities in its phase 1 trial, limiting its therapeutic index and clinical development [27]. We have recently developed a novel EphA2-targeted antibody directed nanotherapeutic (ADN), named EphA2-ILs-DTXp (MM-310), which encapsulates a hydrolytically sensitive prodrug of docetaxel (DTXp) [30]. EphA2-ILs-DTXp was engineered to have advantageous pharmacokinetic properties, resulting in decreased plasma exposure to DTX while maintaining selective tumor exposure [30]. Preclinical studies suggest that EphA2-targeting contributes to EphA2-ILs-DTXp activity primarily through a shift in the microdistribution of the drug, resulting in increased tumor area drug exposure [30].

Here, we report the results of studies characterizing EphA2 expression in bladder cancer. We surveyed EphA2 expression in both primary and metastatic disease, evaluated its prognostic value, and tested the in vivo activity of EphA2-ILs-DTXp (as a monotherapy and in combination with gemcitabine) in EphA2-positive patient-derived xenograft (PDX) models of bladder cancer. Our data highlight the prognostic value of EphA2 in bladder cancer as well as the therapeutic potential of the EphA2-targeting antibody-directed nanotherapeutic EphA2-ILs-DTXp, supporting its clinical exploration as a novel targeted therapy for the treatment of urothelial carcinoma.

## 2. Materials and Methods

### 2.1. Histology and EphA2 Immunohistochemistry

A highly annotated bladder cancer tissue microarray (TMA) was generated at Roswell Park Cancer Institute. Clinical data delivery and Honest Broker services for this study were provided by the Clinical Data Network, which is funded by the National Cancer Institute, grant P30CA016056. Surgical resections and biopsies were acquired from various vendors including Conversant Bio (Huntsville, AL, USA) and Avaden (Seattle, WA, USA). Immunohistochemistry (IHC) for EphA2 was performed on a Dako Link48 instrument (Agilent Technologies, Santa Clara, CA, USA) or LabVision Autostainer360 (Thermo Fisher Scientific, Waltham, MA, USA). Briefly, sections were deparaffinized, antigens were retrieved by heating just below boiling in Tris-EDTA buffer for 25 min in a PT Link (Agilent Technologies, Santa Clara, CA, USA). Endogenous peroxidases and nonspecific binding were blocked by sequential 10 min incubations with Peroxidazed 1 and Background Sniper (Biocare Medical, Concord, CA, USA), following which specimens were probed with a rabbit monoclonal anti-EphA2 antibody (Clone D4A2, Cell Signaling Technology, Danvers, MA, USA). Bound antibody was detected with Envision Plus (Agilent Technologies, Santa Clara, CA, USA) and visualized using DAB.

Images were acquired using an Aperio AT Turbo scanning microscope (Leica Biosystems, Wetzlar, Germany). EphA2 scoring of tumor cells and tumor-associated blood vessels in whole sections was performed by a board certified pathologist. Normal adjacent tissue was also evaluated for EphA2 staining using the same staining protocol. In summary, tumor cells were scored by evaluating the percentage of positive cells as well as the intensity of the signal. Tumor-associated blood vessels, defined as any vessel located within 2 mm of any tumor cell, were scored based on the number of high-power fields (40× objective, 0.25 mpp) that included at least one positive blood vessel. A sample was considered EphA2 positive for tumor-associated blood vessels if 2 or more high power fields of view were positive. Given the smaller size of the tissue areas in TMAs, cores were blindly scored based on a combination of staining intensity and number of EphA2+ cancer cells by two independent diagnostic scientists. Triplicate samples were included in the TMA for each patient and an average of the score between the replicates was used. PD-L1 was also stained and scored in serial sections according to a previously developed protocol [31], using rabbit monoclonal anti-PD-L1 clone E1L3N (Cell Signaling Technology).

### 2.2. Nanotherapeutic (EphA2-ILs-DTXp) Generation

EphA2-ILs-DTXp was prepared as described by Kamoun [30]. Briefly, the anti-EphA2 scFv protein 310-24 scFv was expressed in mammalian cell culture, purified by protein-A affinity chromatography, and conjugated through an engineered C-terminal cysteine residue to the maleimide-terminated lipopolymer, mal-PEG-DSPE. The 310-24 scFv was previously described as scFv-3 by Geddie and has a T_m_ of 71.7 °C and a monovalent K_D_ of 4.1 nM [32]. EphA2-ILs-DTXp was then prepared as previously described [30]. Liposomes were prepared with a lipid composition of egg sphingomyelin/cholesterol/PEG-DSG at a molar ratio of 3:2:0.18. Liposomes were initially formed by extrusion through polycarbonate membranes of 0.1 µm pore size using 1.1 N triethylammonium sucroseoctasulfate as the hydration solution (Dr. Reddy’s; Hyderabad, India). An electrochemical gradient was subsequently generated through removal of the extraliposomal salt using tangential flow filtration. Then, 2′-O-(4-diethylamino butanoyl) docetaxel prodrug (DTXp) was loaded into the liposomes at elevated temperature and trapped inside the liposomes by forming a stable complex with sucroseoctasulfate. Targeting ligand scFv (24)-PEG-DSPE was inserted into the drug-loaded liposomes by micellar insertion at 60 °C for 30 min at a ratio of 5 g conjugate/mol phospholipid. Finally, liposomes were exchanged to the formulation buffer (5 mM citrate, 250 mM NaCl, pH 5.5) by TFF, sterile filtered, and stored at 4 °C. There were two lots of EphA2-ILs-DTXp used in the subsequent studies, with lot TC0215 having a DTXp concentration of 6.02 mg/mL, a phospholipid (PL) concentration of 16.14 mM, DTXp-to-PL ratio of 373.1 g/mol, particle size of 113.6 nm, polydispersity (PDI) of 0.038, and purity of 98.95% DTXp and 1.05% DTX. Lot TG2015 had a DTXp concentration of 6.32 mg/mL, a PL concentration of 16.08 mM, DTXp-to-PL ratio of 387.3 g/mol, particle size of 103.5 nm, PDI of 0.030, and purity of 98.70% DTXp and 0.78% DTX. The concentration and purity of DTX and DTXp were determined by HPLC, the particle size was measured using dynamic light scattering using a Malvern Zetasizer Nano, and the phospholipid concentration was determined using an inorganic phosphate assay.

### 2.3. PDX Models BL-0382, BL-0293, and BL-0440

All experiments and animal husbandry were performed according to animal study protocols approved by the Merrimack Pharmaceuticals Institutional Animal Care and Use Committee (Ethical Number: MAP-014/2017). Murine tolerability studies followed a strict dose escalation protocol, with daily monitoring of the overall health status and measurement of body weight 2–3 times per week. Morbidity criteria included a decrease in animal body weight of more than 20% and/or deterioration in the overall health condition. In addition to overall morbidity criteria, tumor-bearing animals were humanely euthanized by CO_2_ asphyxiation if the tumor volume reached a maximum size of 2000 mm^3^ or showed cavitation and/or ulceration.

Ten-week old female NOD.Cg-Prkdcscid Il2rgtm1Wjl/SzJ (NSG) mice engrafted with either BL-0293, BL-0382, or BL-0440 PDX models were acquired from The Jackson Laboratory. The source and patient characteristics for all the PDX models utilized is shown in Table S1. The PDX tumors were finely minced and approximately 40 µL of material was inoculated subcutaneously in the right flank using a 14 gauge x ¾” needle on a 1 cc syringe. Animals were engrafted no more than 2 weeks prior to shipment and allowed to acclimate after shipment for 1–2 weeks. Tumors were monitored until the average tumor volume was approximately 150 mm^3^. The passage number was less than 10, and the engraftment efficiency was 100%, for all three models. Mice were then staged into groups of eight mice with similar tumor volume distributions prior to treatment. Animals received i.v. lateral tail vein injections of saline, EphA2-ILs-DTXp at 59 mg/kg, or DTX at 10 mg/kg. Gemcitabine was dosed i.v. at 150 mg/kg (BL-0382 and BL-0440) or 75 mg/kg (BL-0293) followed by EphA2-ILs-DTXp at 29 mg/kg 6 h after the gemcitabine injection. Animals were dosed once weekly for four weeks. Body weights and tumor volumes were monitored twice weekly.

### 2.4. PDX Model BL417362

The experiment with model BL417362 was conducted at Northwestern University Developmental Therapeutics Core Facility according to Northwestern University Institutional Animal Care and Use Committee guidelines, under approved animal protocols. Female 4–6-week-old NSG mice from Jackson Laboratory were engrafted with tumors and monitored twice weekly for tumor growth. As the tumors exceeded 150 mm^3^ in volume, mice were enrolled into one of eight treatment arms, with enrollment completed over the course of 10 weeks due to heterogeneous tumor growth rates. Animals were treated once weekly for four weeks by lateral tail vein injections under surgical plane anesthesia maintained with inhaled 2.5–3.5% isoflurane. Dose groups included the following: saline, EphA2-ILs-DTXp at 59 mg/kg, docetaxel at 10 mg/kg, gemcitabine at 150 mg/kg followed by EphA2-ILs-DTXp at 29 mg/kg 6 h after the gemcitabine injection, and gemcitabine at 150 mg/kg followed by DTX at 5 mg/kg 6 h after the DTX injection. Body weight and tumor volumes were monitored twice weekly. Formalin-fixed paraffin embedded samples of all models were provided by The Jackson Laboratory and Northwestern University for EphA2 screening prior to the experiment.

### 2.5. In Vivo Activity

After inoculation of cells, palpable growth was monitored until tumors reached an average volume of 150–300 mm^3^, and the tumor-bearing mice were then randomized into treatment groups. Study animals were treated once weekly for up to four weeks with intravenous tail injections of chemotherapeutic nanoparticles or saline (control). Tumor volume (TV) was monitored twice weekly and was calculated using TV = 0.5 × (L) × (W)^2^ where L is the longest tumor axis and W is the shortest tumor axis measured with calipers. Mice dosed with agents resulting in reduced tumor volumes were monitored until tumor regrowth (120–200 days). In addition to plotting single tumor growth curves to enable unbiased visualization of the data, anti-tumor activity was assessed by calculating maximal tumor regression (100 × (TV_min_–TV_d0_)/TV_d0_, with statistical analysis), or counting the days required for the tumor to reach a size double its volume at day 0 (Time for regrowth (TTR)). We used Kaplan–Meier analysis, which allows partial inclusion of animals that were sacrificed prior to size doubling either related to termination of the study or related to any morbidity criteria including tumor ulceration or body weight loss, analyzed statistically using log-rank Chi-square. Kaplan–Meier analysis was also used to assess morbidity, which can be related to any of four factors: greater than 20% decrease in animal body weight, tumor cavitation and/or ulceration, tumor volume reaching maximum size of 3000 mm^3^, or overall health conditions meeting morbidity criteria.

### 2.6. Statistical Analysis

The data are presented as means with standard errors. ANOVA and posthoc Tukey HSD were used to analyze statistical significance between groups in terms of maximal tumor regression. Categorical analysis with LR Chi-square and Pearson Chi-square were used to analyze statistical significance between groups in terms of regression type. Log-rank and Wilcoxon Chi-square were used to analyze time to regrowth and survival. All statistical analyses were performed in JMP 11 software (SAS, Cary, NC, USA).

## 3. Results

### 3.1. Expression Pattern of EphA2 in Bladder Cancer

**Figure 1:**
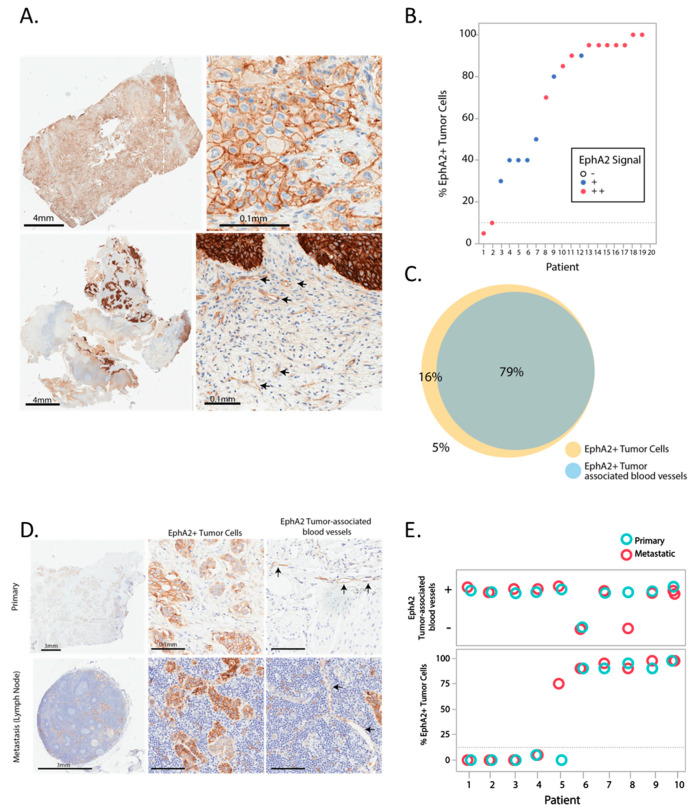
Expression pattern of EphA2 in bladder cancer. (**A**) Micrographs illustrating the expression of EphA2 in bladder cancer patient samples stained with an EphA2 specific immunohistochemistry (IHC) antibody. The top panels illustrate expression in tumor cells shown in low and high magnification, illustrating a cell membrane pattern of staining. The bottom panels illustrate expression at low and high magnification in tumor cells and tumor-associated blood vessels (arrows). (**B**) Quantification of the percentage of EphA2+ tumor cells and EphA2 expression magnitude in 20 urothelial carcinoma samples. (**C**) Prevalence of EphA2 expression in tumor cells and tumor-associated blood vessels in 20 urothelial carcinoma samples. (**D**) Representative EphA2 IHC micrographs of paired primary and metastasis bladder cancer samples from the same patient illustrating a consistent pattern of EphA2 staining in tumor cells and tumor-associated blood vessels. (**E**) Quantification of the percentage of EphA2+ tumor cells and EphA2+/− tumor-associated blood vessels in 10 matched metastases primary samples.

EphA2 expression was initially assessed in twenty surgical resections of urothelial carcinomas by IHC. EphA2 was expressed in cancer cells primarily on the cell membrane, but was also observed in tumor-associated blood vessels (Figure 1A). EphA2 was detected in ≥10% of cancer cells (EphA2-positive) in 19 of 20 samples (95%), and was detected in tumor-associated blood vessels in approximately 80% of the samples (Figure 1B,C). Only 1 of 20 patient samples showed EphA2 in <10% of cancer cells (5%). There was a strong concordance between expression in cancer cells and expression in tumor-associated blood vessels. All samples that had EphA2-positive tumor-associated blood vessels showed also EphA2 expression in ≥10% of cancer cells (Figure 1C). EphA2 expression in ten matched pairs of primary and metastatic lesions of urothelial carcinomas (Table 1) showed a high level of concordance (90% concordance) in EphA2 expression in tumor cells and tumor-associated blood vessels between primary and metastatic lesions (Figure 1D,E). In contrast, normal adjacent tissue in the bladder showed little or no staining for EphA2 (Figure S1).

### 3.2. Correlation of EphA2 Expression with Overall Survival in Bladder Cancer

**Figure 2:**
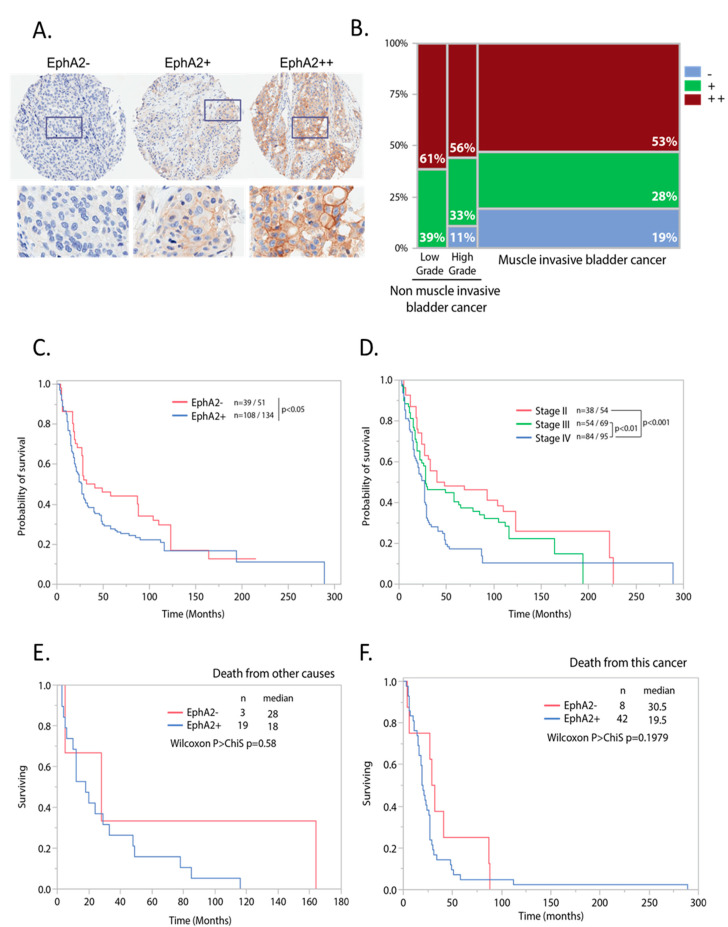
EphA2 is expressed with high prevalence and correlates with poorer survival in bladder cancer. EphA2 was determined using IHC on the expanded set of 177 IHC samples. (**A**) Examples of EphA2 IHC scores in human tissues (EphA2−, EphA2+, EphA2++). Top panels show the full tissue microarray (TMA) cores (diameter 0.5 mm) and the bottom panel shows higher magnification views (width 0.16 mm). (**B**) Prevalence of EphA2 in tumor cells relative to histological grade. (**C**) Kaplan–Meier curve for overall survival independent of cause of death in patients grouped by EphA2 status (**D**) Kaplan–Meier survival curve for overall survival in patients grouped by tumor stage. (**E**) Kaplan–Meier curve for overall survival for death from other causes in patients grouped by EphA2 status. (**F**) Kaplan–Meier curve for overall survival for death from other causes in patients grouped by EphA2 status. Number of patient (*n*), and median survival (median) per group are inserted.

To expand our analysis of EphA2 prevalence, EphA2 expression was assessed in a tissue microarray with 177 urothelial carcinoma samples, including 48 non-muscle invasive and 129 muscle invasive bladder cancer of various stages. Tumor-associated blood vessels were not assessed because of the small analyzable tissue area. Samples were considered EphA2-positive if ≥10% of cancer cells were stained positive for EphA2, irrespective of staining intensity. Positive samples were further characterized as EphA2+ or EphA2++ based on signal intensity. Figure 2A shows tumor cell EphA2 expression assessed with examples of tissues scored EphA2−, EphA2+, and EphA2++ based on signal intensity and percentage of positive tumor cells. EphA2 expression ranged from 80–100% depending on the histological grade of the tumor, with the highest prevalence in low grade non-muscle invasive bladder cancer, followed by high grade non-muscle invasive bladder cancer, and the lowest prevalence (81% positive samples) in muscle invasive tumors (Figure 2B). These samples (*n* = 119; 10 samples were not evaluable due to low tumor content) were used to investigate the relationship between EphA2 expression and overall survival for patients with muscle invasive bladder cancer. For this analysis, we focused on EphA2− vs. EphA2+ by considering both EphA2+ and EphA2++ as EphA2-positive. Patients with EphA2-positive tumors had shorter overall survival with a median of 87 vs. 27 months, respectively; however, this difference did not reach statistical significance (Figure 2C—Wilcoxon ChiSquare *p* = 0.09). As expected, advanced American Joint Committee on Cancer (AJCC) histological stage did correlate with poor prognosis (Figure 2D). Further analysis of the data by cause of death did not change the findings regarding EphA2’s potential prognostic effect (Figure 2E,F). Evaluation of co-expression with PD-L1 identified 44.2% of patients as PD-L1+/EphA2+, while 37.5% were PD-L1−/EphA2+. This analysis included the 119 samples of non-muscle invasive bladder cancer previously used for the survival analysis.

### 3.3. Efficacy of EphA2-Targeted Antibody-Directed Nanotherapeutic in Patient-Derived Models of Bladder Cancer

**Figure 3:**
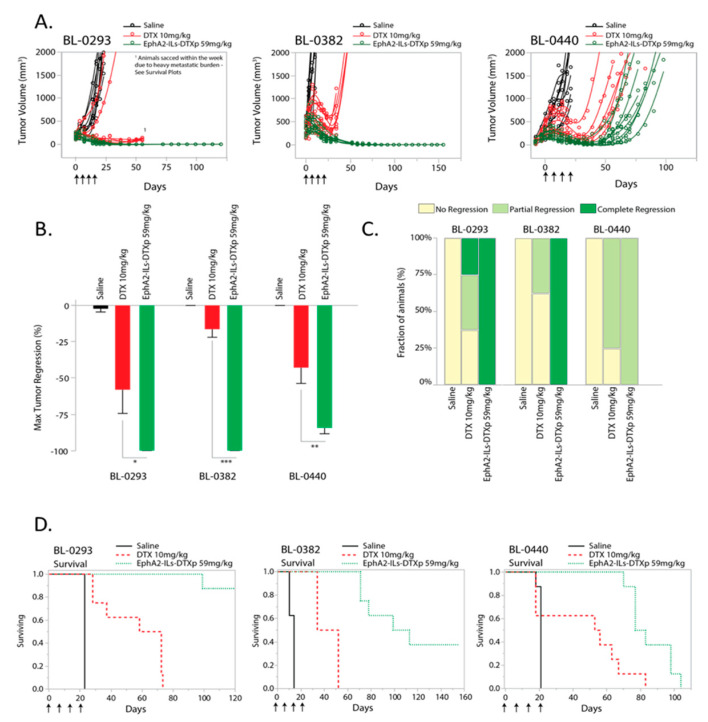
EphA2-targeted docetaxel-based antibody directed nanotherapeutic (ADN) (EphA2-ILs-DTXp) is active in three patient-derived xenograft (PDX) models of bladder cancer. (**A**) Single tumor growth curves of tumor-bearing animals treated with four weekly doses of saline (black), docetaxel (DTX) at 10 mg/kg (red), or EphA2-ILs-DTXp at 59 mg/kg (green), both dose levels are equivalent to the 50% maximum tolerated dose tested in non-tumor bearing animals (*n* = 8 mice). (**B**) Kaplan–Meier analysis of survival for BL-0293, BL-0382, and BL-0440 when comparing EphA2-ILs-DTXp vs. DTX, Log-Rank Prob > ChiSq: BL-0293 *p* < 0.001, BL-0382 *p* < 0.0001, BL-440 *p* < 0.01. (**C**) Maximum tumor regressions induced by DTX or EphA2-ILs-DTXp. ANOVA and post-hoc Tukey HSD * *p* < 0.05, ** *p* < 0.01, *** *p* < 0.0001 (**D**) Regression type analysis performed by computing the number of animals in each tumor regression type.

We recently developed a novel EphA2-targeted ADN (EphA2-ILs-DTXp). This formulation encapsulates a docetaxel pro-drug (DTXp), which converts to active docetaxel (DTX) upon release from the nanotherapeutic. To test the activity of EphA2-ILs-DTXp in EphA2-expressing bladder cancer, we performed in vivo experiments in mice carrying subcutaneous bladder cancer tumors. We used three EphA2-positive PDX models of bladder cancer (Figure S2), and we compared EphA2-ILs-DTXp to equitoxic dosing of free docetaxel (Figure 3 and statistical summary—Table 2), as previously determined in naïve mice [30]. Animals were treated weekly for four weeks either with 59 mg/kg of EphA2-ILs-DTXp or the equitoxic dose of 10 mg/kg of DTX. In all three models, EphA2-ILs-DTXp led to significant tumor regression that extended beyond the treatment period (Figure 3A). Significant improvements in antitumor activity were observed when compared with saline or free docetaxel (ANOVA and post-hoc Tukey HSD EphA2-ILs-DTXp vs. DTX; BL-0293 *p* < 0.05; BL-0382 *p* < 0.001; BL-0440 *p* < 0.01) (Figure 3B). In the BL-0293 and BL-0382 tumor models, EphA2-ILs-DTXp induced durable complete regressions in most treated animals, while free docetaxel led to only partial tumor regressions (Figure 3C). The BL-0440 tumor model was more resistant and led to partial regressions for both EphA2-ILs-DTXp and free docetaxel (Figure 3C).

To assess the magnitude and durability of response, we monitored tumors for an extended period of time, ranging from 100–120 days post-initiation of treatment, and determined the time-to-tumor regrowth and mouse survival. In BL-0293 and BL-0382 tumor models, shorter survival was not only attributed to tumor regrowth, but was driven by unexplained weight loss, possibly caused by the metastatic progression that was mainly seen in the DTX treated group and partially seen in the EphA2-ILs-DTXp group (Figure 3D). Independent of cause of morbidity, EphA2-ILs-DTXp extended survival significantly compared to free DTX (Log-Rank Prob>ChiSq EphA2-ILs-DTXp vs. DTX; BL-0293 *p* < 0.0001; BL-0382 *p* < 0.0001; BL-0440 *p* < 0.01). Together, these results suggest that liposomal encapsulation and EphA2 targeting affect exposure of the drug in tumor cells, leading to a pronounced and sustained in vivo activity.

### 3.4. EphA2-ILs-DTXp Synergizes with Gemcitabine in PDX Models of Bladder Cancer

Gemcitabine is one of the standard of care drugs for the treatment of metastatic bladder cancer [33,34]. Therefore, we tested the activity of EphA2-ILs-DTXp in combination with gemcitabine and compared it to either drug alone. Animals bearing three EphA2-positive PDX models of bladder cancer (BL-0293, BL-0382, BL-0440) were treated with four weekly doses of gemcitabine, EphA2-ILs-DTXp, or their combination. In all models, a subtherapeutic dose of 29 mg/kg was used for EphA2-ILs-DTXp to enable combination analysis. Gemcitabine was given at a lower dose (75 mg/kg) for the gemcitabine sensitive BL-0293 model, whereas for the other two models it was dosed at 150 mg/kg. The BL-0293 bladder PDX model was previously shown to have very low levels of MDM2, which is known to reactivate TP53, and of RB1, a tumor suppressor gene involved in regulating the cell cycle and the tumor’s response to DNA damage caused by cytotoxics like gemcitabine [35].

**Figure 4:**
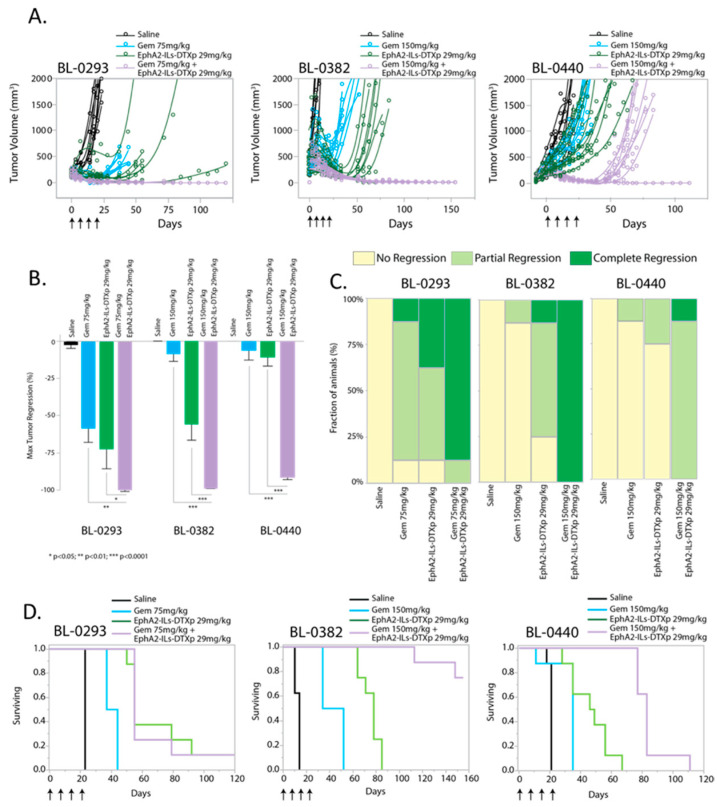
EphA2-targeted docetaxel-based ADN (EphA2-ILs-DTXp) is active in combination with gemcitabine in three PDX models of bladder cancer. (**A**) Single tumor growth curves of tumor-bearing animals treated with four weekly doses of saline (black), gemcitabine (Gem) (cyan), EphA2-ILs-DTXP (green), or combination (magenta) (n = 8 mice for all groups). (**B**) Kaplan–Meier analysis of survival for BL-0293, BL-0382, and BL-0440 when comparing Combination vs. Gem, Log-Rank Prob > ChiSq: all models *p* < 0.0001, Combination vs. EphA2-ILs-DTXp Log-Rank Prob > ChiSq: BL-0382 *p* < 0.0001, BL-440 *p* < 0.0001. (**C**) Maximum tumor regressions induced by Gem, EphA2-ILs-DTXp, or combination. ANOVA and post-hoc Tukey HSD * *p* < 0.05, ** *p* < 0.01, *** *p* < 0.001. (**D**) Regression type analysis performed by computing the number of animals in each tumor regression type.

In all three tested models, the combination of EphA2-ILs-DTXp and gemcitabine led to significant tumor responses that extended beyond the treatment period (Figure 4A). The combination of EphA2-ILs-DTXp and gemcitabine led to significantly greater tumor regression than either drug alone (ANOVA and post-hoc Tukey HSD Combination vs. Gem: BL-0293 *p* < 0.01; BL-0382 *p* < 0.0001; BL-0440 *p* < 0.0001; Combination vs. EphA2-ILs-DTXp: BL-0293 *p* < 0.05; BL-0382 *p* < 0.0001; BL-0440 *p* < 0.0001) (Figure 4B). The combination of EphA2-ILs-DTXp and gemcitabine led to complete tumor regressions in almost all animals in both BL-0293 and BL-0382, which were superior to either drug alone (Figure 4C). In BL-0440, the combination induced only partial tumor regressions but was still superior to the monotherapy arms (Figure 4C). Since BL-0440 was found to be more resistant to taxanes than the other two models, we also tested the combination of gemcitabine with a higher dose of EphA2-ILs-DTXp (59 mg/kg) (Figure S3). High dose EphA2-ILs-DTXp in combination with gemcitabine was able to induce durable and complete regressions in most tested animals. In BL-0382 and BL-0440, survival was significantly extended in the combination arm vs. the monotherapy arms (Log-Rank Prob > ChiSq Combination vs. Gem: BL-0382 *p* < 0.0001; BL-0440 *p* < 0.0001; Combination vs. EphA2-ILs-DTXp: BL-0382 *p* < 0.0001; BL-0440 *p* < 0.0001) (Figure 4D). However, in the BL-0293 PDX model, the combination did not extend survival beyond the effect of EphA2-ILs-DTXp monotherapy, suggesting that the effects of adding gemcitabine were limited (Log-Rank Prob > ChiSq Combination vs. Gem: BL-0293 *p* < 0.0001; Combination vs. EphA2-ILs-DTXp: BL-0293 *p* = 0.97) (Figure 4D).

### 3.5. EphA2-ILs-DTXp in Combination with Gemcitabine is Superior to Free Docetaxel in Combination with Gemcitabine

**Figure 5:**
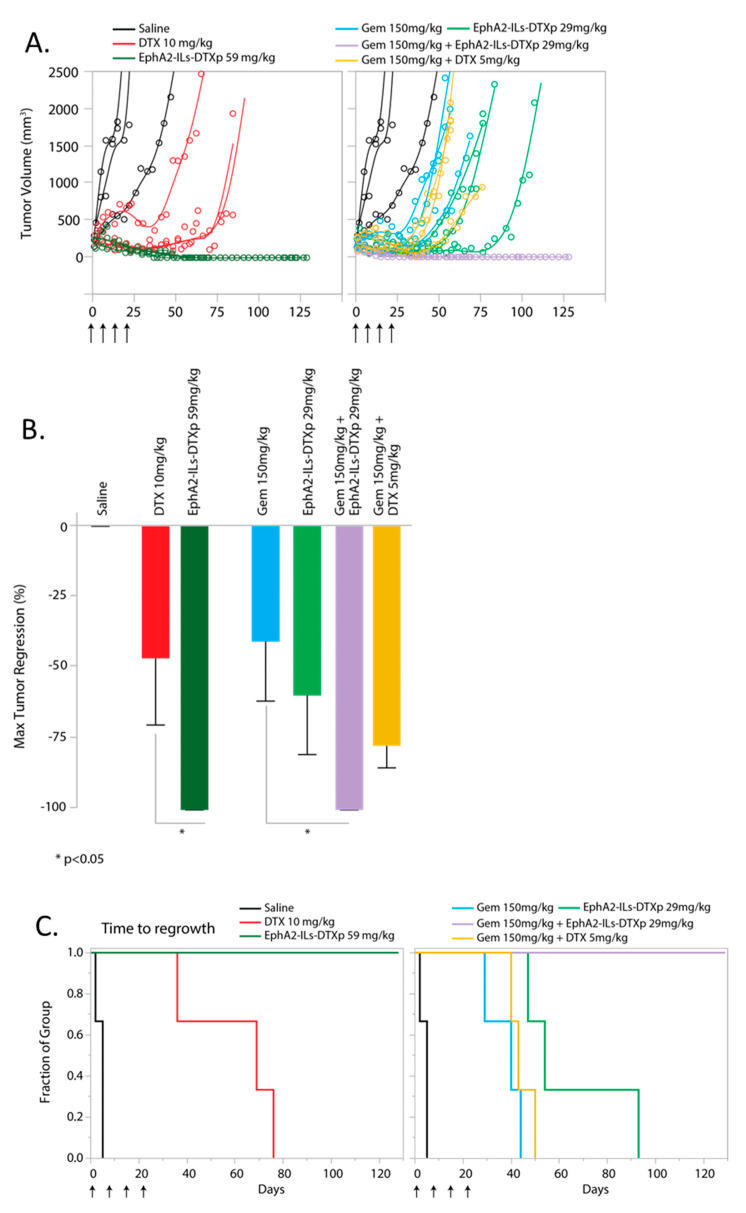
EphA2-targeted docetaxel-based ADN (EphA2-ILs-DTXp) in combination with gemcitabine is more active than the equitoxic dose of docetaxel combined with gemcitabine (*n* = 3). (**A**) Single tumor growth curves of tumor-bearing animals treated with four weekly doses of saline (black), Gem (cyan), DTX (red), EphA2-ILs-DTXp (dark/light green), Gem/EphA2-ILs-DTXp (magenta), or Gem/DTX combination (yellow). (**B**) Kaplan–Meier analysis of time to regrowth for BL417362 when comparing EphA2-ILs-DTXP 59 mg/kg vs. DTX 10 mg/kg, Log-Rank Prob > ChiSq: *p* < 0.05, EphA2-ILs-DTXp/Gem vs. DTX/Gem Log-Rank Prob > ChiSq: *p* < 0.05. (**C**) Maximum tumor regressions induced by Gem, DTX, EphA2-ILs-DTXp, or combinations. ANOVA and post-hoc Tukey HSD * *p* < 0.05.

The activity of EphA2-ILs-DTXp in combination with gemcitabine was compared to the combination of equitoxic dosing of free docetaxel with gemcitabine in an additional EphA2-positive bladder cancer model (BL417362) (Figures S1D and S5). Statistical power analysis suggested that an *n* = 3 would be sufficient to test the hypothesis assuming that the response in this model was similar to the previous models. Animals were treated with four weekly doses of drugs either in monotherapy or combination arms and were followed until tumor regrowth after treatment. The combination of EphA2-ILs-DTXp at 59 mg/kg and gemcitabine led to durable and complete tumor regressions in all animals (Figure 5A). Consistent with the other models, EphA2-ILs-DTXp at 59 mg/kg led to significant tumor regression with extended survival when compared with an equitoxic dose of free DTX at 10 mg/kg (ANOVA and post-hoc Tukey HSD; *p* < 0.05; Log-Rank Prob > ChiSq Survival: *p* < 0.05) (Figure 5B). EphA2-ILs-DTXp in combination with gemcitabine induced significantly more tumor regression than gemcitabine monotherapy (Figure 5B) (ANOVA and post-hoc Tukey HSD; Combination vs. Gem: *p* < 0.05) and led to a significant extension of survival when compared to either drug alone or to equitoxic combination of gemcitabine and free DTX (Figure 5C) (Log-Rank Prob > ChiSq Survival: EphA2-ILs-DTXp/Gem vs. Gem *p* < 0.05; EphA2-ILs-DTXp/Gem vs. EphA2-ILs-DTXp *p* < 0.05; EphA2-ILs-DTXp/Gem vs. DTX/Gem *p* < 0.05).

## 4. Discussion

Despite a number of significant advances in the treatment of metastatic bladder cancer, the development of new therapies remains an area of important unmet medical need. Here, we show that EphA2 is an attractive target for molecular targeting in bladder cancer, and we demonstrate the preclinical activity of an EphA2-targeted ADN. ADNs can be considered a next-generation format of antibody drug conjugates, in which the active drug is linked only indirectly to the antibody targeting ligand through a larger nanocarrier that encapsulates the drug [30]. For the treatment of bladder cancer and other solid tumors, we developed an EphA2-targeted ADN encapsulating a novel docetaxel prodrug, DTXp. This novel format enables the use of the potent chemotherapeutic docetaxel and does not require the ultra-high potency auristatins or maytansines of conventional ADCs. The design of EphA2-ILs-DTXp enables efficient and specific delivery of a large payload of docetaxel to tumor cells, while reducing toxicity compared to conventional chemotherapy [30]. These particles were effective in multiple EphA2-positive bladder cancer models, either as monotherapy or in combination with gemcitabine. The improved efficacy of EphA2-ILs-DTXp compared to docetaxel is expected to be accompanied by reduced toxicity. Like other liposomes, EphA2-ILs-DTXp confines the drug to the circulatory system; drug sensitive tissues are exposed to less docetaxel, which in turn results in reduced toxicity [30]. Free docetaxel distributes widely throughout the body, and can cause bone marrow, liver, immune, neurological, and other severe toxicities in patients [36]. In mice, these toxicities manifest as weight loss due to lack of appetite or metabolic dysregulation. In our studies, docetaxel caused weight loss in the animals during treatment and shortly after the end of treatment (Figures S4 and S5). In some cases, animals were euthanized due to excessive (>20%) weight loss or poor body condition during treatment. In comparison, EphA2-ILs-DTXp did not cause significant weight loss in the monotherapy setting (Figures S4 and S5).

The EphA2-binding arm is designed to target the extracellular domain of EphA2, enhancing retention and delivery in the tumor [30]. It has been hypothesized that targeted liposomes are especially effective in disseminated metastases since the targeting arm improves microdistribution and enables better retention in the tumor [30,37]. The EphA2 expression pattern in bladder cancer suggests that EphA2-ILs-DTXp would efficiently target metastatic urothelial carcinomas. Our analysis of 20 samples of surgically resected bladder cancer shows that the vast majority of tumors overexpressed EphA2 on more than 10% of tumor cells and that the majority of these urothelial carcinomas also express EphA2 on the tumor vasculature. EphA2 expression does not change in metastases; in 9 out of 10 matched samples concordant expression was observed, and in the one non-concordant sample, EphA2 expression was gained in the metastatic lesions. The concordant expression extends to tumor-associated vasculature, where there was concordant expression in 9 out of 10 samples, with one metastatic sample lacking EphA2 expression on the vasculature. These data suggest that selection of EphA2-positive cancer patients for treatment with EphA2-ILs-DTXp may be accomplished by measurement of the EphA2 using IHC in the primary surgical resections rather than in fresh biopsies. This will be confirmed in the clinic, where initial clinical trials will also look at the concordance between fresh biopsies and primary tissue.

Our survey of patient tumor samples supports the prognostic relevance of EphA2 expression in urothelial carcinomas. Although not statistically significant, there was a strong trend for shorter survival for patients with EphA2-positive tumors compared to patients with negative tumors. This result is consistent with reports in the literature. In particular, high EphA2 expression in malignant tumors has been shown to be associated with poor prognosis in NSCLC, hepatocellular carcinoma, and breast cancer [38,39].

Platinum-based chemotherapy has been the standard of care for more than a decade in metastatic bladder cancer, with overall survival ranging from nine to fifteen months [40,41]. The second- and third-line therapies have been evolving rapidly, with a series of targeted agents being approved in recent years, most notably checkpoint inhibitors [9,11]. The high mutational burden observed in bladder cancer [42] makes it an attractive target for both PD-1 and PD-L1 inhibitors, since cancers with high mutation burdens show higher response rates [43,44]. Additionally, atezolizumab and pembrolizumab are also first-line treatment options for patients with locally advanced or metastatic urothelial cell carcinoma not eligible for cisplatin-containing chemotherapy regimens [9,10]. We have also observed promising activity for EphA2-ILs-DTXp in combination with immune checkpoint inhibitors in syngeneic cancer models [45]. Thus, EphA2-ILs-DTXp could represent a promising combination partner not only with other chemotherapies, but also with anti-PD1/PD-L1 drugs in bladder cancer. Clinical characterization of such combinations in the clinical setting is warranted.

EphA2 is a promising target for the treatment of bladder cancer, since this receptor is strongly expressed in both primary and metastatic tumors. Our work demonstrates that targeting EphA2 in bladder cancer with the EphA2-ILs-DTXp ADN produces significantly better results than docetaxel alone. Furthermore, combinations of the ADN with gemcitabine produce synergistic activity, demonstrating that EphA2-ILs-DTXp could be a valuable clinical option. EphA2-ILs-DTXp (MM-310) is currently undergoing clinical evaluation to determine the toxicity profile and preliminary efficacy potential of the drug in patients with advanced solid tumors (NCT03076372).

## Figures and Tables

**Table 1:** Tumor location and EphA2 staining in patients with recurrent disease.

Patient ID	Resection Site	% EphA2+ Tumor Cells	EphA2 TAV
110056928	Bladder	0	+
	Lymph Node	0	+
110060015	Bladder	90	+
	Cervix	97.5 ^1^	+
110066840	Bladder	90	+
	Lymph Node	95	+
110067477	Bladder	0	+
	Lymph Node	75	+
110068115	Bone	75	+
110068802	Bladder	0	+
	Soft Tissue	0	+
110069061	Bladder	5	+
	Lymph Node	5	+
110071199	Bladder	0	+
	Lymph Node	0	+
110071455	Bladder	97.5 ^1^	+
	Lymph Node	97.5 ^1^	+
	Uterus	97.5 ^1^	+
110076214	Bladder	95	+
	Seminal Vesicle	90	-

^1^ Pathologist score 95–100.

**Table 2:** Statistical analysis of the in vivo activity of EphA2-ILs-DTXp in PDX models.

**Monotherapy**							
**PDX Model**	**Treatment Group-**	**n**	**Max Regression**			**Survival**	
			**mean**	**std**	***p***	**median**	***p***
BL-0293	DTX 10 mg/kg	8	−58	46	*p <* 0.05	65	*p <* 0.0001
	EPhA2-ILs-DTXp 59 mg/kg	8	−100	0		Not reached	
BL-0382	DTX 10 mg/kg	8	−16	16	*p <* 0.001	43	*p <* 0.0001
	EPhA2-ILs-DTXp 59 mg/kg	8	−100	0		106	
BL-0440	DTX 10 mg/kg	8	−43	31	*p <* 0.01	54.5	*p <* 0.01
	EPhA2-ILs-DTXp 59 mg/kg	8	−84	12		80	
BL417362	DTX 10 mg/kg	3	−47	41	*p <* 0.05	83	*p <* 0.05
	EPhA2-ILs-DTXp 59 mg/kg	3	−100	0		Not reached	
**Gemcitabine Combination**							
**PDX Model**	**Treatment Group**	**n**	**Max Regression**			**Survival**	
			**mean**	**std**	***p*^1^**	**median**	***p*^1^**
BL-0293	Gem 75 mg/kg	8	−58	26	*p <* 0.01	41	n.s.
	EPhA2-ILs-DTXp 29 mg/kg	8	−72	37	*p <* 0.05	55	n.s.
	EPhA2-ILs-DTXp + Gem	8	−99	2		55	
BL-0382	Gem 150 mg/kg	8	−9	14	*p <* 0.0001	43	*p <* 0.0001
	EPhA2-ILs-DTXp 29 mg/kg	8	−57	30	*p <* 0.0001	78	*p <* 0.0001
	EPhA2-ILs-DTXp + Gem	8	−100	0		Not reached	
BL-0440	Gem 150 mg/kg	8	−6	18	*p <* 0.0001	35	*p <* 0.0001
	EPhA2-ILs-DTXp 29 mg/kg	8	−11	16	*p <* 0.0001	48	*p <* 0.0001
	EPhA2-ILs-DTXp + Gem	8	−93	4		83	
BL417362	Gem 150 mg/kg	3	−41	36	*p <* 0.05	59	*p <* 0.05
	EPhA2-ILs-DTXp 29 mg/kg	3	−60	36	n.s.	83	*p <* 0.05
	DTX + Gem	3	−77	13	n.s.	59	*p <* 0.05
	EPhA2-ILs-DTXp + Gem	3	−100	0		Not reached	

^1^ All comparisons were done vs. EphA2-ILs-DTXp + Gem; n.s.: not significant.

## Supplementary Materials

The following are available online at https://www.mdpi.com/1999-4923/12/10/996/s1, Figure S1. EphA2 expression in bladder normal adjacent tissue. EphA2 expression is limited to tumor area (left of dotted line), while normal adjacent tissue shows very little EphA2 expression by IHC. Scale bar 100 µm Figure S2. EphA2 Expression in PDX bladder models. EphA2 is expressed on tumor cell membranes in the four selected PDX models of bladder cancer, visualized by immunohistochemistry. Scale bar 100 µm Figure S3 EphA2-ILs-DTXp dosed at 59 mg/kg combined with gemcitabine at 150 mg/kg leads to regression of the resistant model: BL-440. (A) Single tumor growth curves of tumor-bearing animals treated with four weekly doses of saline (black), Gem (cyan), EphA2-ILs-DTXp (green), Gem/EphA2-ILs-DTXp (magenta) (B) Kaplan-Meier analysis of survival. Log-Rank Prob > ChiSq comparing Combination vs. EphA2-ILs-DTXp *p* < 0.01, Combination vs. Gem: *p* <0.0001. Figure S4. Change in body weight in PDX model BL-0293. Single animal body weight curve for all the treatment groups illustrating the decrease in body weight loss detected in the DTX-treated animals Figure S5. Change in body weight in PDX model BL417362. Single animal body weight curve for all the treatment groups illustrating the decrease in body weight loss detected in the DTX-treated animals as monotherapy, and when combined with gemcitabine. Table S1. Origins of patient-derived xenograft models employed.
