# Supplementary material for: Targeting EphA2 in Bladder Cancer Using a Novel Antibody-Directed Nanotherapeutic
Source: Pharmaceutics. 2020 Oct 20;12(10):996. doi: 10.3390/pharmaceutics12100996 (PMC7589819; doi:10.3390/pharmaceutics12100996)
Supplement: Supplementary file 1 [file pharmaceutics-12-00996-s001.pdf]

pharmaceutics

MDPI

Supplementary Materials

Targeting EphA2 in Bladder Cancer Using a Novel Antibody-Directed Nanotherapeutic

Supplementary Materials: The following are available online at www.mdpi.com/xxx/s1, Figure S1: title, Table S1: title, Video S1: title.

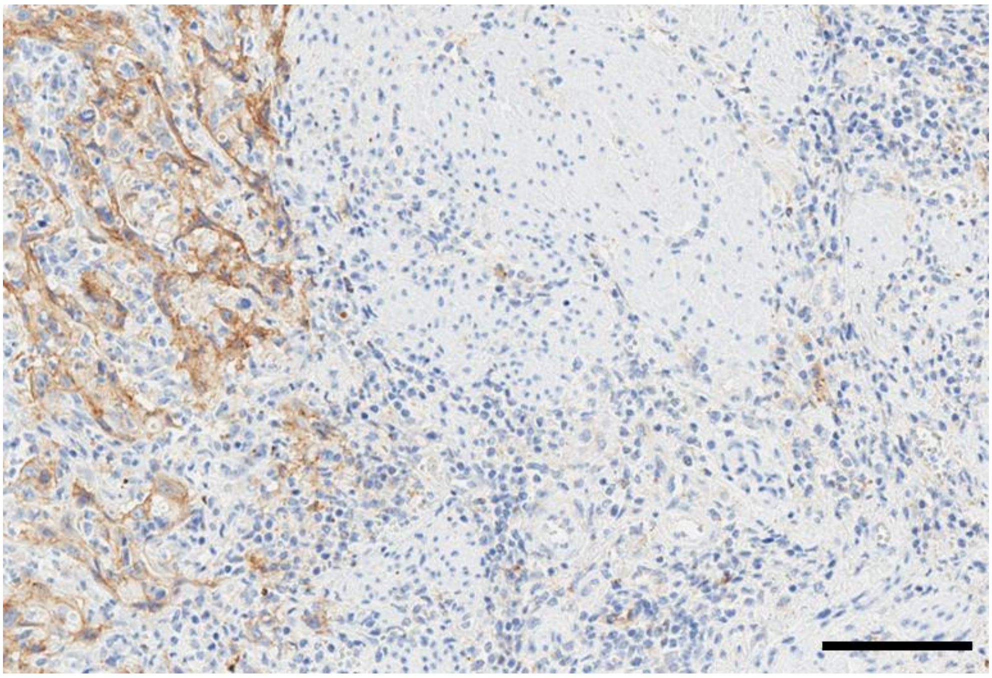

Supplemental Figure S1. EphA2 expression in bladder normal adjacent tissue. EphA2 expression is limited to tumor area (left of dotted line), while normal adjacent tissue shows very little EphA2 expression by IHC. Scale bar 100 μm

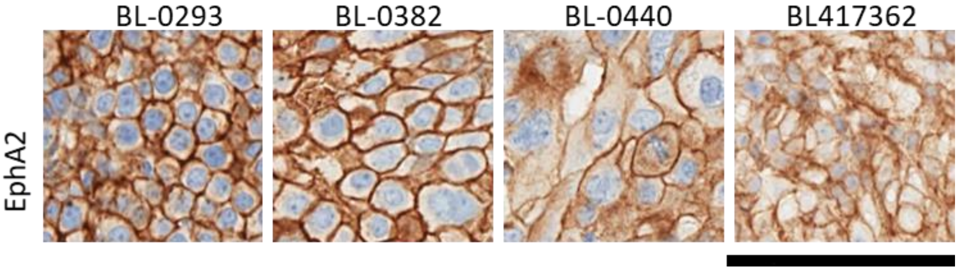

Supplemental Figure S2. EphA2 Expression in PDX bladder models. EphA2 is expressed on tumor cell membranes in the four selected PDX models of bladder cancer, visualized by immunohistochemistry. Scale bar 100 μm

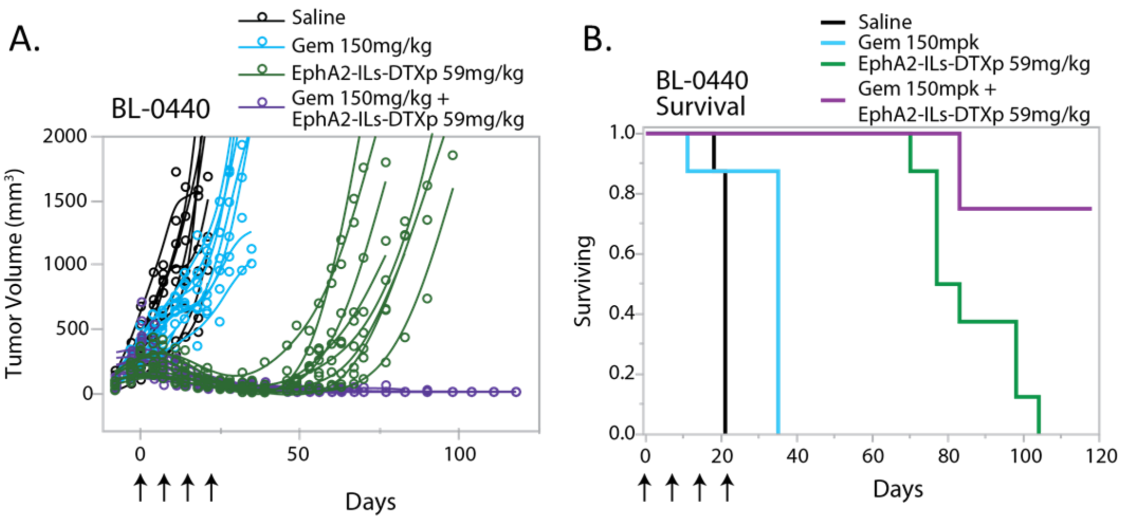

Supplemental Figure S3. EphA2-ILs-DTXp dosed at 59 mg/kg combined with gemcitabine at 150 mg/kg leads to regression of the resistant model: BL-440. (**A**) Single tumor growth curves of tumor-bearing animals treated with four weekly doses of saline (black), Gem (cyan), EphA2-ILs-DTXp (green), Gem/EphA2-ILs-DTXp (magenta) (**B**) Kaplan-Meier analysis of survival. Log-Rank Prob > ChiSq comparing Combination vs. EphA2-ILs-DTXp $p < 0.01$, Combination vs. Gem: $p < 0.0001$.

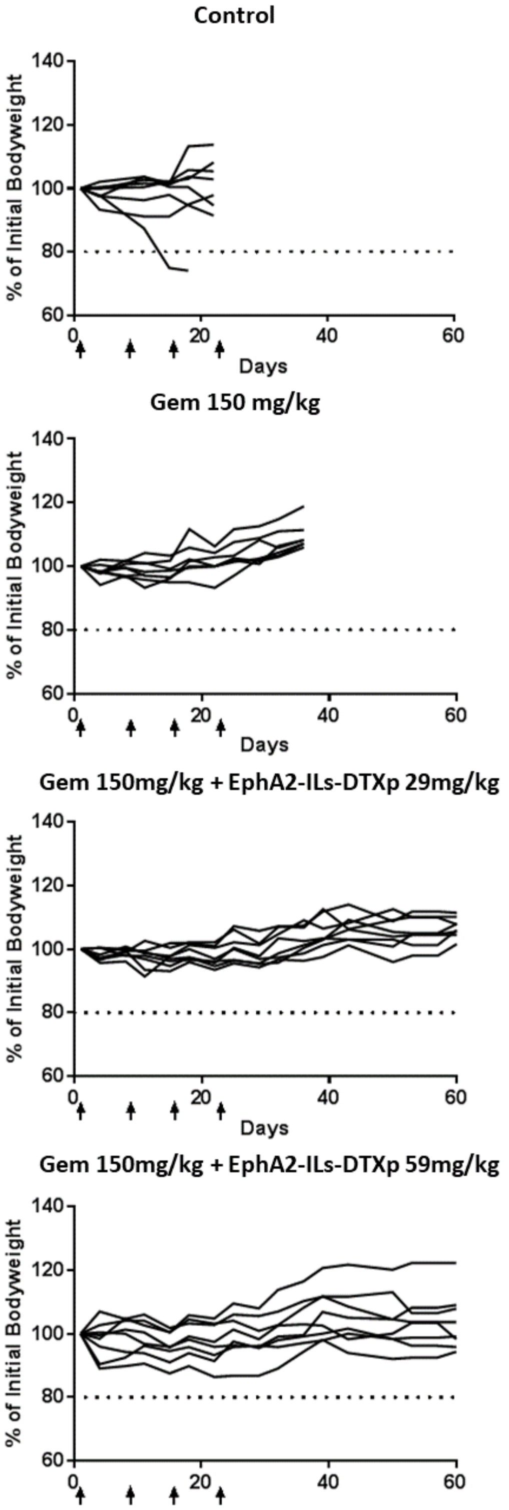

Supplemental Figure S4. Change in body weight in PDX model BL-0293. Single animal body weight curve for all the treatment groups illustrating the decrease in body weight loss detected in the DTX-treated animals.

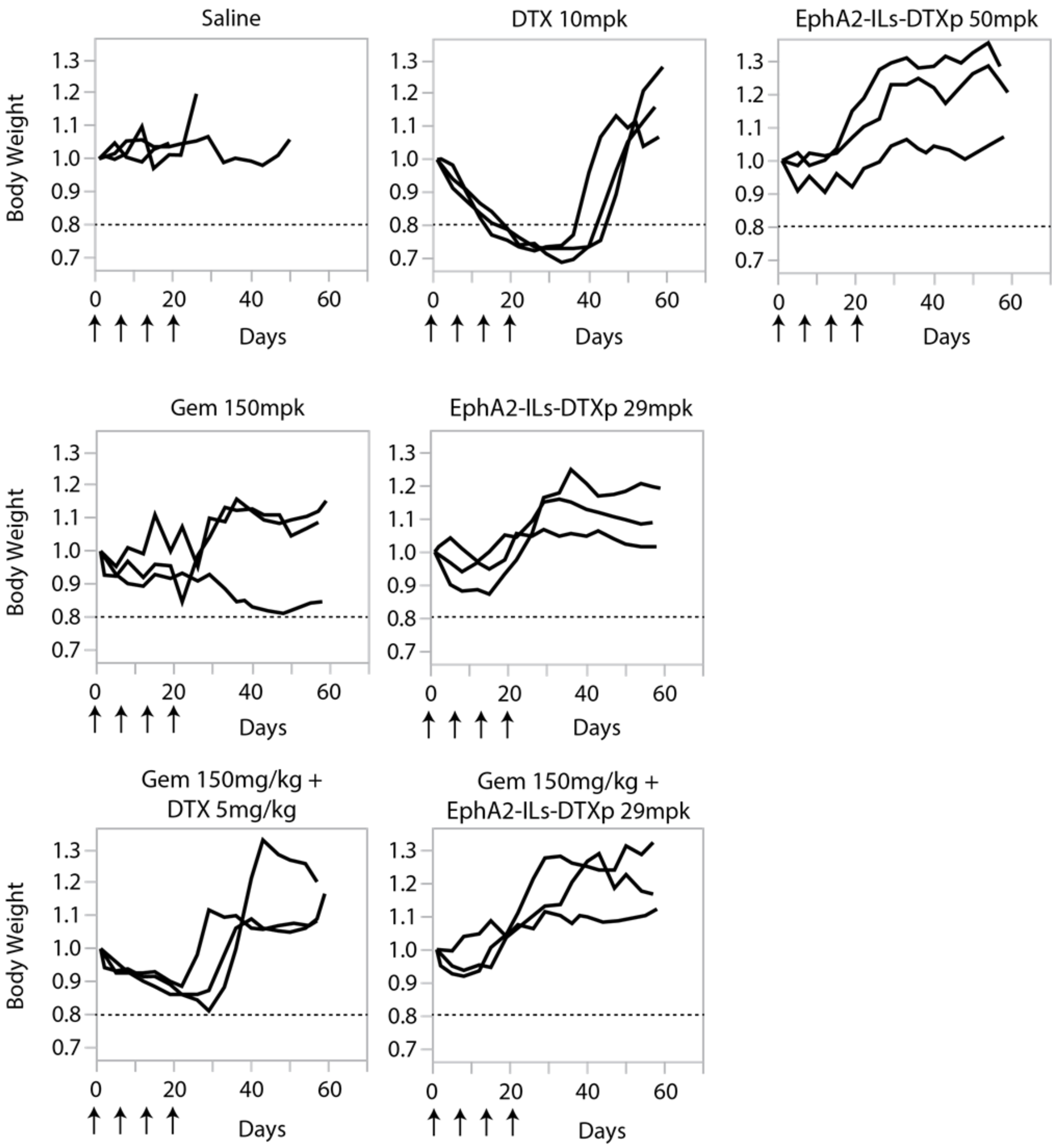

Supplemental Figure S5. Change in body weight in PDX model BL417362. Single animal body weight curve for all the treatment groups illustrating the decrease in body weight loss detected in the DTX-treated animals as monotherapy, and when combined with gemcitabine.

Supplemental Table S1. Origins of patient-derived xenograft models employed.

PDX ID	Diagnosis	Grade	Path Stage	Stage	Tissue Type	Sex	Age	Source of Models
BL0923F	Urothelial carcinoma	High	pT2a N2 Mx	IV	Cystectomy	F	77	Jackson Laboratories
BL0382F	Urothelial carcinoma	High	pT2 High	III	TURBT	F	82	Jackson Laboratories
BL0440F	Urothelial carcinoma	High	pT4a N2 Mx	IV	Cystectomy	M	71	Jackson Laboratories
BL-417362	Urothelial carcinoma	NA	NA	NA	Cystectomy	M	77	Northwestern University

NA; not available.

© 2020 by the authors. Submitted for possible open access publication under the terms and conditions of the Creative Commons Attribution (CC BY) license (http://creativecommons.org/licenses/by/4.0/).
